# Refining O-RADS MRI: beyond malignancy risk

**DOI:** 10.1007/s00330-025-12038-6

**Published:** 2025-10-17

**Authors:** Sebastian Harth, Ivo Meinhold-Heerlein

**Affiliations:** 1 Department of Diagnostic and Interventional Radiology, University Hospital Gießen, Justus Liebig University of Gießen, Gießen, Germany; 2 Department of Gynecology and Obstetrics, University Hospital Gießen, Justus Liebig University of Gießen, Gießen, Germany

Transvaginal ultrasound is considered the primary imaging technique for the evaluation of adnexal masses, while MRI is utilized as an additional technique to characterize indeterminate lesions and to reduce false-positive findings that may result in unnecessary surgery. In order to standardize imaging, reporting, and subsequent management of adnexal lesions, Andreotti et al introduced the Ovarian-Adnexal Reporting and Data System (O-RADS) for ultrasound in 2020 [1]. The American College of Radiology (ACR) O-RADS MRI Committee subsequently introduced the O-RADS MRI scoring system in 2021 [2] and provided guidelines on the application in clinical practice in 2022 [3].

While the O-RADS MRI risk stratification system is intended primarily to estimate the probability of malignancy in adnexal lesions, further differentiation may be clinically useful in practice, considering the various histopathological subtypes of ovarian tumors [4]. Particularly, patients who wish to conceive prefer fertility-conserving surgery. Thus, treatment decisions with major consequences may depend on the presumed histopathology of adnexal masses (e.g., ovarian cystectomy vs salpingo-oophorectomy or bilateral vs unilateral salpingo-oophorectomy) [5]. It is therefore extremely valuable to identify additional MRI criteria to distinguish different histological subtypes of adnexal lesions and to allow individual and appropriate patient management.

In this issue of *European Radiology*, Florin and colleagues [6] report a retrospective multi-center study evaluating the value of imaging criteria in combination with the O-RADS MRI score for the prediction of histopathological diagnoses of adnexal masses, including 869 adnexal lesions from 651 patients. The data originate from the EURAD (EURopean ADnexal study) database, including patients from 15 European centers [7].

The authors present comprehensive analyses of the influence of imaging, clinical, and laboratory factors on the probability of histopathological diagnoses of adnexal lesions. In O-RADS 4 lesions, papillary projections and a mean ADC value of > 1.08 × 10⁻³ mm²/s were identified as independent predictive factors of borderline tumors (AUC 0.89; 95% CI: 0.83–0.94). In O­-RADS 5 lesions, the presence of mural nodules, an ADC value of solid tissue of < 1.08 × 10⁻³ mm²/s, menopause, an elevated CA-125 level, and a mean lesion size >80 mm were identified as independent predictive factors of primary invasive tumors (AUC 0.89; 95% CI: 0.83–0.92). In O-RADS 4 and 5 lesions, a completely solid composition was an independent predictor of metastases (AUC 0.79; 95% CI: 0.73–0.84). Other key observations were the significance of fatty components and multiple different signal intensities in T1- and T2-weighted sequences for the diagnosis of mature cystic teratomas, endometriotic fluid, and low ADC values of cystic components (< 2.0 × 10⁻³ mm²/s) for the diagnosis of endometriomas; and the presence of > 10 cystic loculi for the diagnosis of benign mucinous cystadenomas.

While some well-known associations between imaging features and certain subtypes of adnexal masses are now confirmed, how might patient management change with the addition of histopathological subtype prediction to the O-RADS MRI risk stratification? First, appreciating papillary projections and assessing the ADC values of solid portions of adnexal masses to differentiate between invasive tumors (exhibiting lower ADC values) and borderline tumors (exhibiting higher ADC values), in addition to the O-RADS score, may alter the surgical staging approach. While hysterectomy and bilateral salpingo-oophorectomy are usually performed during surgical staging procedures of ovarian cancer, the hysterectomy is not mandatory, and a fertility-conserving approach can be offered for the non-invasive borderline tumors—even though the probability of recurrence is higher when one or both of the ovaries remain. Second, the imaging prediction of metastasis rather than a primary invasive ovarian tumor in cases of adnexal masses with a completely solid composition can change patient management and shift the focus from the ovary to the primary tumor. Third, it may be useful to consider indicators of histology in cases of almost certainly benign lesions or low risk lesions (O-RADS category 2 or 3) like cystadenomas, although differences in the therapeutic approach to serous and mucinous tumors are particularly important in relation to the malignant counterparts of these entities, with mucinous tumors typically confined to early stages and showing a low rate of occult lymph node metastasis [8, 9].

While the combination of the O-RADS MRI score with the data presented by Florin and colleagues may provide guidance for refining the diagnostics of adnexal lesions, future efforts should include validation in larger collectives. Although the cohort studied is not small, some histopathological diagnoses are only represented to a limited extent (e.g., 40 borderline tumors and 35 benign mucinous cystadenomas). Another important future step would be to provide guidance and algorithms for use in clinical practice, which is a major strength of the O-RADS MRI risk stratification system, enabling general radiologists to perform at a level similar to that of subspecialty radiologists [3].
